# Acute TRPTI™ (oleoylethanolamide) supplementation enhances incretin hormone responses: a fixed-sequence crossover study

**DOI:** 10.3389/fendo.2026.1879799

**Published:** 2026-07-15

**Authors:** David Briskey, Janice Pellow, Pavitra Viswanath, Amanda Rao

**Affiliations:** 1 RDC Clinical, Brisbane, QLD, Australia; 2 Gencor Pacific Ltd, Hong Kong, Hong Kong SAR, China

**Keywords:** DPP-4, GIP, GLP-1, incretin hormones, insulin, OEA, oleoylethanolamide, TRPTI

## Abstract

**Background:**

Oleoylethanolamide (OEA) is an endogenous lipid mediator involved in nutrient sensing and gut-derived hormonal signalling. TRPTI™ is a bioavailable OEA formulation designed to support metabolic and appetite-related pathways. This study evaluated the acute metabolic effects of TRPTI™ in healthy adults under controlled feeding conditions.

**Methods:**

In this fixed-sequence, single-blind, placebo-controlled, three-period crossover study, 37 adults (BMI 25–34.9 kg/m²) received a single dose of a placebo, 150 mg TRPTI™, or 300 mg TRPTI™ following an overnight fast. Blood samples were collected over 8 hours, with standardised meals provided at 15 minutes and 4 hours post dose. Time-course responses of GLP-1, GIP, DPP-4, insulin, glucose, and glucagon were analysed.

**Results:**

Baseline adjusted GLP-1 and GIP AUC analyses demonstrated significant treatment effects across multiple pre-specified meal-related periods. The 300 mg TRPTI™ dose produced the greatest incretin responses and was significantly greater than placebo during selected pre-specified post-dosing and post-prandial periods, with elevations detectable within 15 minutes of ingestion. At key timepoints, GLP-1 concentrations were significantly higher (approximately 20–25%) than placebo. Similar significant increases were observed for GIP. Significant concentration changes in circulating DPP-4 were observed at later time points, indicating a potential association between TRPTI™ supplementation and serum DPP-4 modulation. Both TRPTI™ doses showed acute tolerability with safety biomarkers remaining within normal ranges. A statistically significant reduction in meal consumption was observed in both TRPTI™ groups.

**Conclusions:**

A single 300 mg dose of TRPTI™ enhances post-meal GLP-1 and GIP responses and demonstrates rapid engagement of GLP-1, GIP and DPP-4, supporting further investigation of its role in nutritional strategies targeting metabolic health.

**Clinical trial registration:**

https://clinicaltrials.gov/study/NCT06840080, identifier NCT06840080.

## 1 Introduction

N-acylethanolamides, or fatty acid ethanolamides, constitute a group of endogenous bioactive lipid molecules formed from both saturated and unsaturated fatty acid precursors. Oleoylethanolamide (OEA) is a bioactive lipid mediator that is a member of the N-acylethanolamine and acylglycerol families. Dietary sources of OEA include oatmeal, nuts, and cocoa powder, however, the amount of OEA found in these foods is relatively low (1). Endogenous OEA is naturally synthesised from cell membranes, specifically membrane glycerophospholipids, primarily in the small intestine, as well as in adipose tissue, neurons, and astrocytes (2). The levels of OEA in the body are dynamically regulated by nutritional status, and its synthesis is dependent on the availability of dietary oleic acid. Dietary consumption of oleic acid through olive oil, nuts and avocados can elevate circulating levels of OEA by increasing the availability of substrates required for OEA biosynthesis. Oleic acid is enzymatically converted into OEA within intestinal enterocytes (3). OEA concentration is reduced in the gut after food deprivation and increases again after eating (2).

Multiple studies have explored the health benefits of OEA supplementation in various population groups. Research suggests OEA offers protective effects against metabolic conditions such as obesity, non-alcoholic fatty liver disease (NAFLD), insulin resistance, and various inflammatory conditions. In individuals with metabolic disorder, several clinical trials have shown OEA improves glycaemic control and lowers serum glucose and insulin levels. It has also been shown to reduce weight, body mass index (BMI), waist circumference and appetite in healthy obese individuals (1).

OEA exerts its biological effects primarily by acting as a high-affinity agonist of the nuclear receptor peroxisome proliferator-activated receptor alpha (PPAR-α), a potent transcription factor that regulates gene expression related to lipid metabolic and inflammatory pathways appetite and glucose homeostasis. PPAR-α receptors are found in the liver, kidneys, heart, small intestine and muscle, and are important regulators of metabolic function (2, 4). OEA acts via PPAR-α receptors to induce fatty acid β-oxidation. It also interacts with transient receptor potential vanilloid 1 (TrpV1), G protein-coupled receptor (GPR)55, and G protein-coupled receptor 119 (GPR119), suggesting complex roles (5). OEA has been shown to delay meal initiation, decrease gastric emptying, enhance lipid metabolism, stimulate incretin hormone production, and suppress various inflammatory pathways including nuclear factor (NF)-κB (2). While the dose of OEA used in clinical trials vary, systematic reviews on the effects of OEA on cardiometabolic factors reported an average daily dose that ranged from 125 to 600 mg (1, 2).

Incretin hormones are peptides released in the gut in response to food intake to counter postprandial hyperglycaemia by stimulating the release of glucose-dependant insulin from the pancreas and inhibiting glucagon secretion. They act to regulate postprandial glucose levels, delay gastric emptying, suppress appetite and enhance satiety (6). The binding of a lipid-derived ligand like OEA to GPR-119 on intestinal L-cells has been shown to potentiate the release of the endogenous incretin hormone Glucagon-Like Peptide-1 (GLP-1), and to a lesser extent Glucose-dependent Insulinotropic Polypeptide (GIP), formerly termed “gastric inhibitory peptide” (5). Both GLP-1 and GIP enhance glucose-dependent insulin secretion from pancreatic β-cells (7, 8). The biological activity of GLP-1 and GIP decreases soon after secretion due to decomposition by DPP-4, an enzyme that breaks down incretin hormones (9). Unlike GLP-1, the effects of GIP on glucagon secretion are glucose dependent. GIP can stimulate glucagon release from pancreatic α-cells during fasting or hypoglycaemic conditions, whereas this effect is diminished or absent when blood glucose concentrations are elevated (10). Despite promising therapeutic potential, the dose-response relationship and mechanisms of OEA’s effects on incretin hormones GLP-1 and GIP remain poorly characterised, limiting optimal dosing strategies for metabolic disorders.

TRPTI™ is a novel supplement that contains a combination of OEA and LipiSperse^®^ technology. LipiSperse^®^ is a cold-water dispersion technology designed to promote the solubility and bioavailability of lipophilic compounds such as OEA and contains no bioactive properties itself. The technology makes use of surfactants, polar lipids, and solvents that embed into OEA’s crystal structure whilst maintaining surface hydrophilicity, increasing water dispersibility and preventing crystal agglomeration. LipiSperse^®^ has previously been shown to improve the bioavailability of the similar compound palmitoylethanolamide (PEA) (11). The combination of OEA with LipiSperse^®^ technology in TRPTI™ aims to optimise OEAs therapeutic efficacy by enhancing its bioavailability.

The aim of this study was to compare the metabolic effects of two different doses of TRPTI™, containing bioavailable OEA and LipiSperse^®^, compared to a placebo in healthy participants over an 8-hour period, with particular focus on incretin hormone modulation. We hypothesised that acute TRPTI™ supplementation would augment meal-stimulated incretin secretion in a dose-dependent manner.

## 2 Materials and methods

### 2.1 Trial design and setting

This single-blind, placebo controlled, fixed-sequence, crossover clinical study was conducted at a single research site in Brisbane, Australia (RDC Clinical), with enrolment from March 2025 to June 2025.

### 2.2 Ethics and trial registration

The study was conducted in accordance with the Declaration of Helsinki and the International Council for Harmonisation Good Clinical Practice (ICH-GCP) guidelines. The protocol and all study procedures were approved by the Bellberry Human Research Ethics Committee (HREC 2024-12-2083-A-1). Written informed consent was obtained from all participants prior to enrolment. The trial was registered at ClinicalTrials.gov (Identifier: NCT06840080).

### 2.3 Participants

Forty-three participants were enrolled who were generally healthy adults aged ≥30 years with a body mass index (BMI) of 25.0–34.9 kg/m² and were able to comply with study procedures. Females were required to use prescribed contraception. The forty eligible participants were medication−free, non−smokers who had no history of neurological, psychiatric, gastrointestinal, or eating disorders and were not adhering to any special dietary regimens. Key exclusion criteria included known glucose or insulin dysregulation or metabolic/endocrine disorders (e.g., diabetes, NAFLD, hyperinsulinemia, hypoglycaemia); serious or unstable illness; use of medications or supplements affecting incretin, glucose, insulin, or satiety pathways; use of OEA- or LipiSperse^®^-containing supplements within 4 weeks; current malignancy (except basal cell carcinoma) or cancer treatment within the previous 2 years; major dietary changes within 1 month; smoking, nicotine use, substance abuse, or excessive alcohol consumption (>21 drinks/week); pregnancy or lactation; known allergy to study ingredients; or any condition that, in the investigator’s opinion, made participation unsuitable.

### 2.4 Interventions

The investigational product was TRPTI™, comprising a standardised, bioavailable oleoylethanolamide (OEA) formulation and Lipisperse^®^. Each TRPTI™ capsule contained 150 mg TRPTI™, delivering ≥125 mg of OEA. Accordingly, the 150 mg TRPTI™ treatment provided ≥125 mg OEA (one active capsule and one placebo capsule), whilst the 300 mg TRPTI™ treatment provided ≥250 mg OEA (two active capsules). Matching placebo capsules contained microcrystalline cellulose, magnesium stearate, and colloidal silica and were identical in appearance to the active product to maintain blinding. Participants received study products in a fixed-sequence: placebo, 150 mg/day TRPTI™, 300 mg/day TRPTI™. All participants consumed two capsules daily, with the 150 mg group receiving one active and one placebo capsule to ensure identical capsule counts across groups. Capsules were taken orally following an overnight fast (≥10 hours) with at least 100 mL of water. Both active and placebo products were manufactured in accordance with Good Manufacturing Practice (GMP) standards in a Therapeutic Goods Administration (TGA)-licensed facility.

### 2.5 Blinding

This study employed a single-blind, placebo-controlled, fixed-sequence, crossover design, with an 8-hour post-dose sampling period in each group. This study design was employed to enable within-subject comparisons, with each participant serving as their own control. Participants completed all three treatments in a predetermined sequence, with a minimum 6-day washout between periods to minimise potential carryover effects. All study products were packaged in identical containers and labelled uniformly to maintain blinding. Participants were blinded to treatment allocation, whilst investigators administering the products were aware of the assigned treatment. The statistician responsible for data analysis remained blinded to group allocation.

### 2.6 Outcomes and assessments

The registered (ClinicalTrials.gov) primary endpoint was change from baseline in GLP-1 AUC over the study period following dosing. Prior to database lock a Statistical Analysis Plan further specified that this endpoint would be evaluated across pre-defined physiologically relevant meal-related periods including: 0–30 min, 45 min-2.5 h, 3–4 h, 5–6 h, 7–8 h and 0–8 h. Secondary outcomes included changes in serum/plasma glucose-dependent insulinotropic polypeptide (GIP), circulating dipeptidyl peptidase-4 (DPP-4), glucagon, glucose, and insulin concentrations; gastrointestinal tolerability assessed via the Gastrointestinal Symptom Questionnaire (12); adverse event monitoring; appetite assessment using a visual analogue scale (VAS); and food intake during clinic visits. Exploratory outcomes included clinical safety markers, including liver function tests and triglycerides which were measured at baseline and at 8h.

### 2.7 Study procedures

To minimise product carryover, participants attended three 8-hour clinic visits in a three-period crossover design, receiving each treatment sequentially (groups 1–3) with a minimum 6-day washout between doses. Participants arrived fasted (≥10 hours), after which baseline anthropometrics and vital signs were recorded and a pre-dose blood sample collected. Two capsules of study product were then administered with water. Serial blood samples were obtained at 0.25–8 hours post-dose. Standardised breakfast and lunch meals were provided at 0.25 and 4 hours, respectively. The standardised breakfast and lunch meals provided approximately 2335 kJ and 3171 kJ, respectively. Macronutrient composition was broadly comparable between meals, with breakfast consisting of 21.7% protein, 21.8% fat, and 56.6% carbohydrates, and lunch consisting of 24.9% protein, 23.2% fat, and 51.9% carbohydrates. Food intake was quantified by expressing the remaining food as percentage consumption. Prior to lunch, participants completed validated gastrointestinal tolerance questionnaires (12) and appetite ratings using a 0–100 mm VAS to assess satiety. Adverse events were monitored throughout each visit.

Serum concentrations of total GLP-1, GIP, DPP-4 (CD26), and glucagon were quantified using commercially available immunoassays (RayBiotech, Peachtree Corners, GA, USA) according to the manufacturer’s instructions. Total GLP-1 was measured using the Human GLP-1 EIA Kit, GIP using the Human GIP EIA Kit, DPP-4 (CD26) using the Human CD26 ELISA Kit, and glucagon using the Human Glucagon ELISA Kit. Samples were analysed using a microplate reader, and concentrations were calculated from assay-specific standard curves generated for each plate. All samples from a participant were analysed on the same plate to minimise inter-assay variability.

### 2.8 Statistical analysis

#### 2.8.1 Sample size

Sample size calculations were performed *a priori* using G*Power (version 3.1) using values from a previously conducted and unpublished pilot study. Assuming a clinically meaningful 40% increase in GLP-1 area under the curve (AUC) between treatments, with a two-sided alpha level of 0.05 and 95% statistical power, a minimum of 33 evaluable participants per treatment condition was required. Power analyses were conducted using a difference between two dependent means (matched pairs), yielding 32 degrees of freedom and a critical t-value of 1.69. Calculations were based on a paired (within-subject) design to reflect the crossover structure of the study and the corresponding reduction in inter-individual variability. To account for potential attrition and incomplete datasets, enrolment was increased to 40 participants per group to ensure adequate power for the primary analysis.

#### 2.8.2 Statistical analysis

All analyses were conducted using R. Data were analysed according to a fixed-sequence, crossover design, with each participant consuming a placebo, 150 mg TRPTI™, and 300 mg TRPTI™ treatments. As the study employed a fixed-sequence crossover design in which all participants received treatments in the same predefined order, sequence effects could not be estimated and were therefore not included in the statistical model. A minimum 6-day washout period was incorporated between treatment periods to minimise potential carryover effects. Hormone concentrations (GLP-1 total, GIP, glucagon, and DPP-4) were baseline-adjusted by subtracting the 0 h value within each subject and treatment. Data are presented as mean ± SEM unless otherwise stated. Unless otherwise specified, the term ‘significant’ refers to statistical significance (p < 0.05).

Incretin hormones such as GLP-1 exhibit highly dynamic, rapidly regulated secretion patterns that are tightly linked to nutrient ingestion. Their physiological role is to provide transient, meal-associated signalling, rather than sustained concentration changes over time. Because of this, summarising the response using a single peak value or an overall AUC can obscure important temporal features of the secretion pattern, particularly during the early post-prandial period. Because incretin responses are transient and closely linked to meal ingestion, baseline-adjusted AUC was calculated over pre-specified periods corresponding to fasting, post-prandial, and inter-meal phases: 0–30 min (post-dose, pre-prandial), 45 min–2.5 h (post-prandial), 3–4 h (pre-prandial), 5–6 h (post-prandial), 7–8 h (pre-prandial) and overall AUC from 0 to 8 hours. The primary analyses focused on pre-specified meal-period AUC responses. Additional time-course analyses were undertaken to further characterise temporal treatment effects.

For the pre-specified meal periods AUC analyses, hormone concentrations were first baseline-adjusted by subtracting the pre-dose (0 h) value from each post-dose timepoint within each participant and treatment condition. Baseline-adjusted area under the concentration-time curve was then calculated using the linear trapezoidal method over each of the pre-specified prandial periods Both positive and negative deviations from baseline were retained in the calculation, such that the resulting AUC values represented the net integrated change over each respective period. Participant-treatment profiles with up to two missing timepoints were retained, and AUC was calculated using available adjacent observations without imputation. Treatment effects on AUC were assessed using repeated-measures one-way ANOVA, with paired *post hoc* comparisons performed where appropriate. Each pre-specified period was analysed independently using repeated-measures one-way ANOVA. Pairwise treatment comparisons were adjusted using Tukey’s multiple-comparison procedure.

In addition to the baseline-adjusted AUC analyses, cumulative AUC was calculated for serum DPP-4 concentrations to characterise the progressive accumulation of treatment effects over time. This approach was not applied to GLP-1 or GIP because these hormones exhibit rapid, transient, and acute meal-responsive secretion patterns, for which cumulative exposure metrics may obscure important temporal treatment effects. Consequently, time-course analyses were used to complement the meal-period AUC analyses and provide additional insight into the temporal pattern of incretin responses.

The primary time-course analysis evaluated treatment effects across the full sampling period using a repeated-measures ANOVA (Outcome = Treatment × Time + Subject), where Treatment and Time were treated as categorical factors and Subject was included as a fixed effect to account for within-subject crossover pairing. When the overall model indicated differences, paired *post hoc* comparisons between treatments were performed across all timepoints (TRPTI™ 300 mg vs placebo, TRPTI™ 150 mg vs placebo, and TRPTI™ 300 mg vs TRPTI™ 150 mg) using Tukey multiple comparison analysis. An adjusted p-value < 0.05 was considered statistically significant. Timepoint-specific pairwise comparisons were interpreted only when a significant overall Treatment × Time interaction was observed.

## 3 Results

### 3.1 Participant demographics characteristics

**Figure 1:**
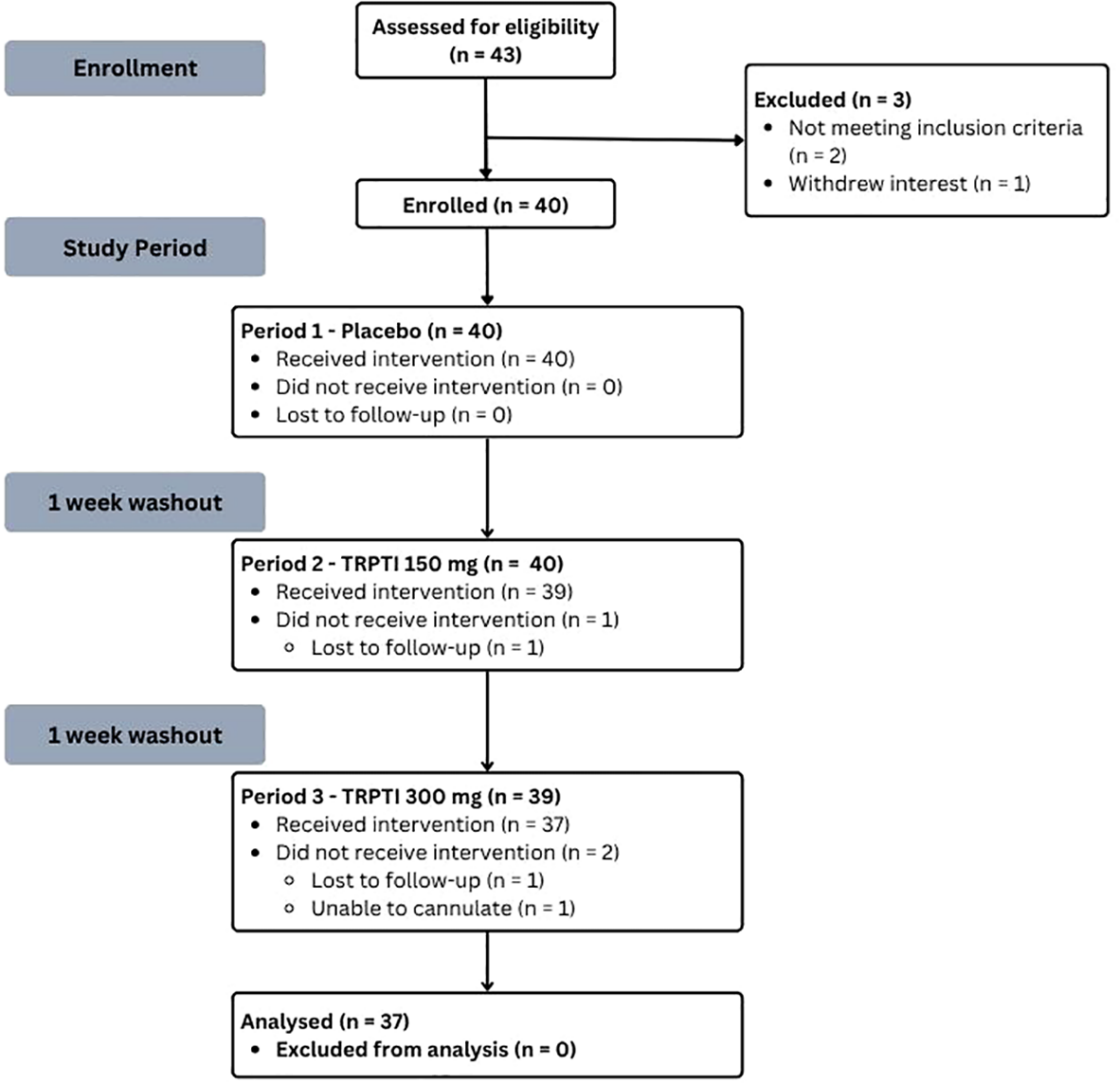
CONSORT flow diagram of participant progress through the trial.

A total of 40 participants were enrolled in the study with 37 completing all requirements and included in analysis (**Figure 1**; **Table 1**). The demographic characteristics of all completed participants (n = 37) are presented in **Table 1**.

**Table 1:** Participant characteristics and demographics (N = 37).

Characteristic	Mean ± SD	Range
Gender (F/M)	23/14	–
Age (years)	50.11 ± 11.36	32-79
Waist (cm)	92.87 ± 9.15	75-110
Hip (cm)	109.21 ± 7.01	93.5-127
Waist-to-Hip Ratio	1.14 ± 0.16	0.71-1.45
Height (m)	1.71 ± 0.124	1.50-1.93
Weight (kg)	82.72 ± 12.01	58.4-115.9
BMI (kg/m^2^)	28.53 ± 3.10	25.02-33.89
Systolic BP (mmHg)	125.73 ± 14.50	97-153
Diastolic BP (mmHg)	79.92 ± 10.37	62-102
Pulse (BPM)	68.11 ± 6.85	54-84

SD = standard deviation.

### 3.2 Glucagon-like peptide-1

Baseline-adjusted GLP-1 AUC differed significantly between groups across several prandial periods, with the pairwise differences varying between periods relative to the groups. During 0–30 min, 300 mg TRPTI™ produced a greater response than both placebo (p=0.012) and 150 mg TRPTI™ (p=0.001; F(2,72)=13.19, p<0.001), whilst the 150 mg group did not differ from placebo (p=0.080). During 45 min–2.5 h, the 150 mg group showed a lower response than both placebo (p=0.002) and 300 mg TRPTI™ (p=0.001; F(2,68)=9.34, p<0.001), with no difference between 300 mg and placebo (p=0.900). During 3–4 h, 300 mg produced a greater response than both placebo (p=0.025) and 150 mg (p=0.002; F(2,68)=6.89, p=0.002), with no difference between 150 mg and placebo (p=0.635). During 5–6 h, 300 mg was greater than 150 mg (p=0.001) and placebo was greater than 150 mg (p=0.041; F(2,68)=8.48, p<0.001), whilst 300 mg did not differ from placebo (p=0.251). During 7–8 h, the 150 mg group showed a lower response than both placebo (p=0.005) and 300 mg (p=0.012; F(2,52)=6.66, p=0.003), with no difference between 300 mg and placebo (p=0.900).

Overall AUC0–8h also differed significantly between groups (F(2,66)=8.68, p=0.0004). Both placebo and 300 mg TRPTI™ groups had a greater integrated response than the 150 mg group (p<0.001 and p=0.003, respectively), with no significant difference between 300 mg and placebo (p=0.162).

**Figure 2:**
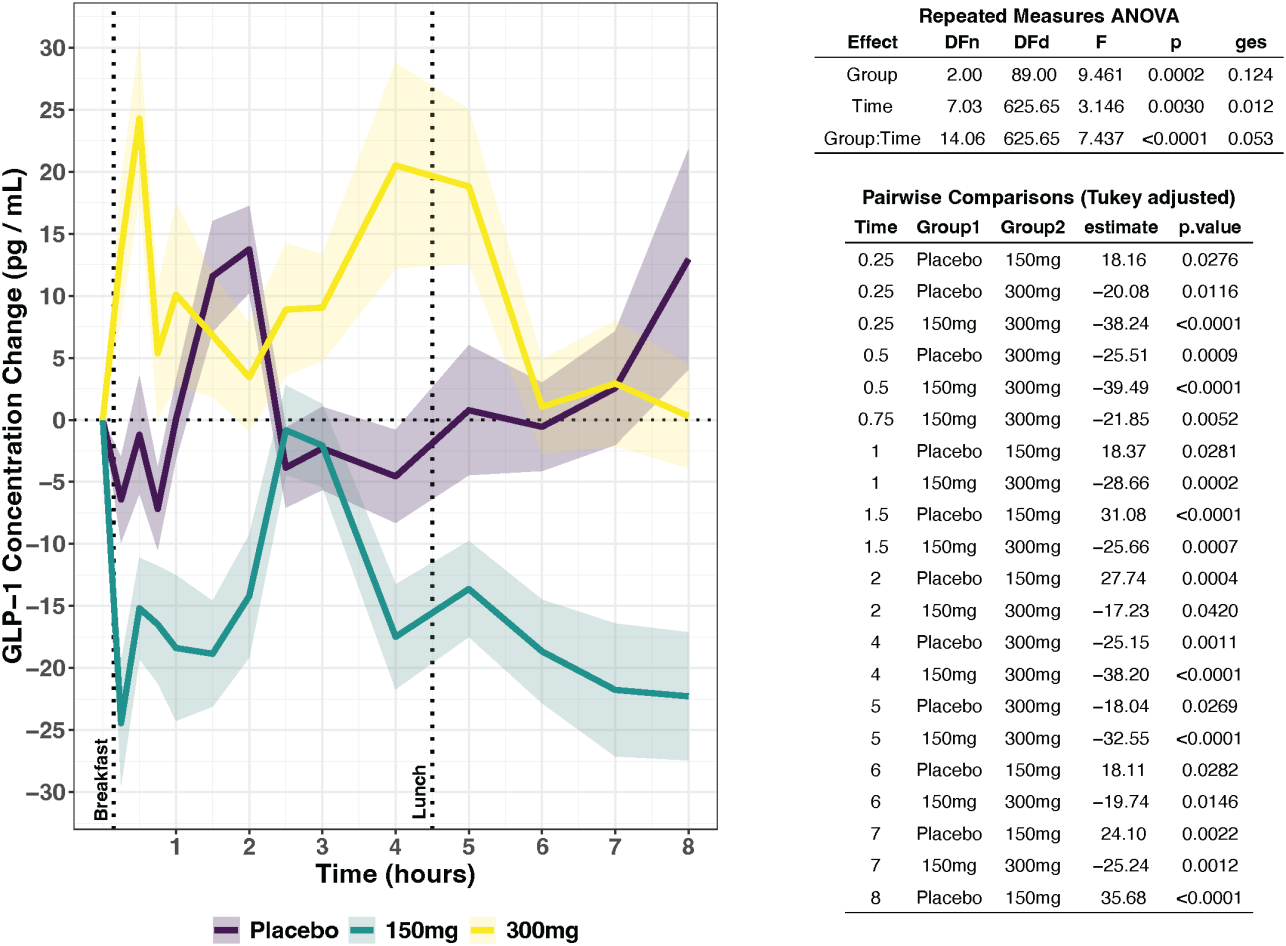
GLP-1 concentration over an 8-hour period following supplementation. Change in concentrations over time. Breakfast, time breakfast was consumed; Lunch, time lunch was consumed.

Time-course analyses revealed treatment-related effects at specific post-prandial intervals. At baseline, GLP-1 concentrations were significantly higher (p < 0.05) in the 150 mg TRPTI™ group compared with both placebo and 300 mg TRPTI™ groups (**Figure 2**; **Supplementary Figure 1**). TRPTI™ produced dose-dependent changes in circulating GLP-1 concentrations over the 8-hour sampling period. A significant main effect of Treatment was observed for baseline-adjusted GLP-1 (p < 0.0001), indicating differences in overall concentrations between treatment conditions. A significant main effect of Time was observed (p = 0.003). A repeated-measures ANOVA revealed a significant treatment × time interaction for GLP-1 responses across the postprandial period (F(14.06, 625.65) = 7.44, p < 0.0001, generalised η² = 0.053), indicating that GLP-1 trajectories differed significantly between treatment conditions over time. Compared with placebo, the 300 mg TRPTI™ dose produced significantly greater increases in total GLP-1 at early post-dose timepoints, with elevations observed predominantly within the first hour following dosing. A second larger GLP-1 peak was observed between 3–6 hours post TRPTI™ consumption, potentially due to nutrient stimulation of L-cells in the ileum. The 150 mg dose showed minimal separation from placebo and did not consistently reach statistical significance, suggesting that 150 mg of TRPTI™ was insufficient to elicit a physiologically meaningful effect on GLP-1. Following supplementation, GLP-1 levels at the post-dose timepoints (0.25 h, 0.5 h and 0.75 h) were significantly higher in the 300 mg TRPTI™ group compared with both the placebo and 150 mg TRPTI™ groups. In addition, GLP-1 levels were also higher between 4–5 hours post TRPTI™ supplementation. No other period showed any significant difference between groups (6–8 h).

### 3.3 Glucose-dependent insulinotropic polypeptide

**Figure 3:**
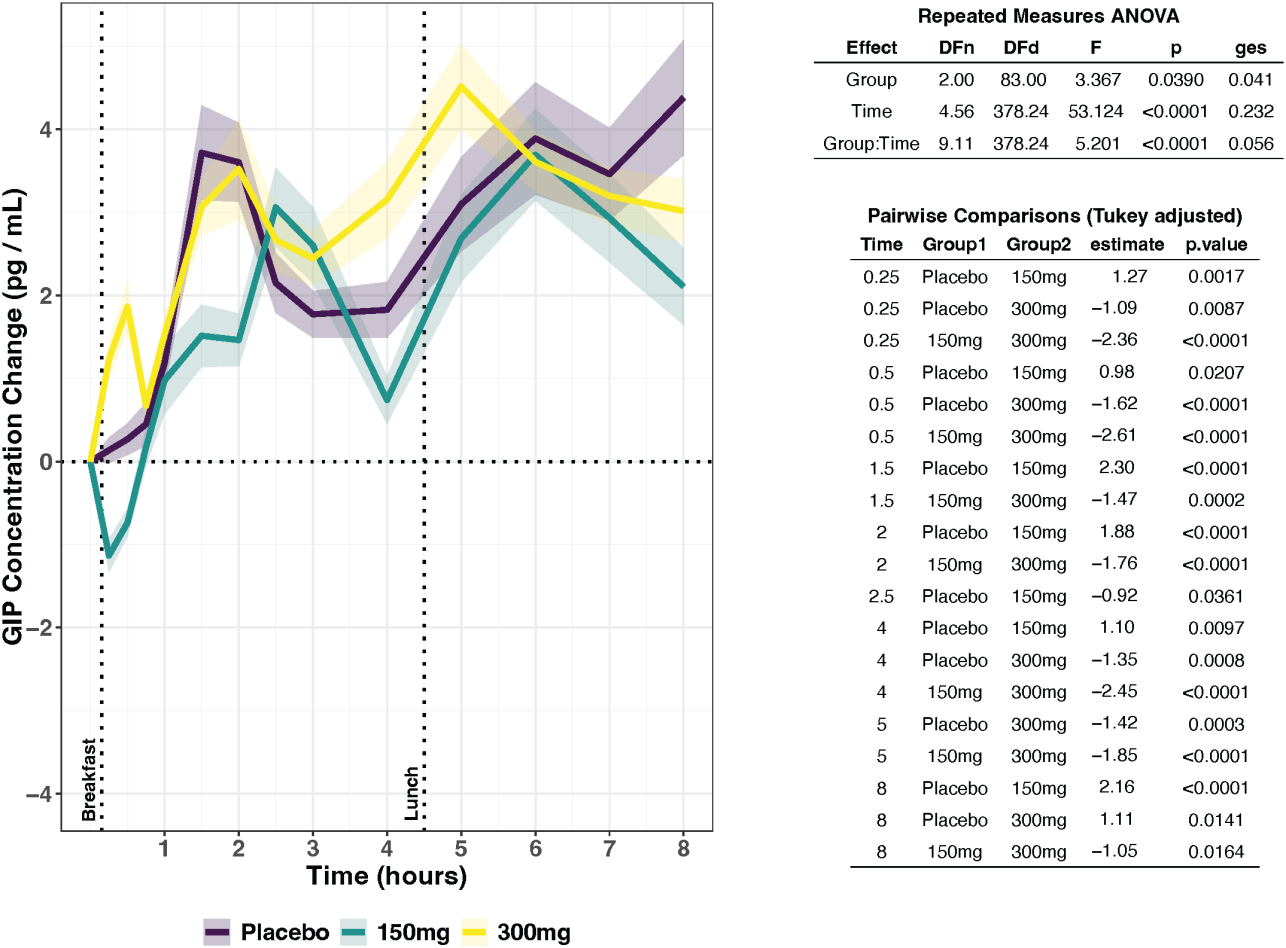
GIP concentration over an 8-hour period following supplementation. Change in concentrations over time. Breakfast, time breakfast was consumed; Lunch, time lunch was consumed.

At baseline GIP concentrations were significantly higher (p < 0.05) in the 150 mg TRPTI™ group compared with both placebo and 300 mg TRPTI™ groups (**Figure 3**; **Supplementary Figure 2**). Baseline-adjusted GIP AUC differed significantly between groups across all prandial periods. During 0–30 min, 300 mg TRPTI™ was greater than both placebo and 150 mg (both p=0.001; F(2,68)=37.35, p<0.001), and 150 mg was lower than placebo (p=0.001). During 45 min–2.5 h, the 150 mg group was lower than both placebo (p=0.001) and 300 mg (p=0.002; F(2,66)=9.86, p<0.001), with no difference between 300 mg and placebo (p=0.900). During 3–4 h, 300 mg was greater than both placebo (p=0.007) and 150 mg (p=0.003; F(2,64)=7.33, p=0.001), with no difference between 150 mg and placebo (p=0.900). During 5–6 h, 300 mg was greater than 150 mg (p=0.027; F(2,68)=3.51, p=0.035), whilst neither active group differed significantly from placebo. During 7–8 h, the 150 mg group was lower than placebo (p=0.004; F(2,50)=5.80, p=0.005), whilst 300 mg did not differ from placebo (p=0.196) or 150 mg (p=0.234).

Overall AUC0–8h differed significantly between groups (F(2,64)=11.27, p<0.001), driven primarily by a higher integrated GIP response in the 300 mg group relative to 150 mg, with comparatively modest differences between placebo and each active group.

A significant main effect of Treatment was observed for baseline-adjusted GIP concentrations (p < 0.05), indicating differences in overall concentrations between treatment conditions. A significant main effect of Time was observed (p < 0.0001). A repeated-measure ANOVA also revealed a significant Treatment × Time interaction (p < 0.0001). GIP concentrations demonstrated significant time-dependent variation consistent with meal-related secretion. The 300 mg dose elicited significantly greater increases in GIP concentrations compared with placebo at early timepoints, whilst the 150 mg dose demonstrated limited or inconsistent effects. A second delayed peak in GIP concentration was also observed in the 300 mg TRPTI™ group compared to placebo between 4–5 hours. Following TRPTI™ supplementation, the initial time point posts breakfast (0.25 h, 0.5 h) showed a significant elevation in GIP in the 300 mg TRPTI™ group compared to both the placebo and 150 mg TRPTI™ groups. Significant GIP elevation was also observed between 4–5 hours post TRPTI™ consumption, corresponding to lunch intake.

### 3.4 Dipeptidyl peptidase-4

Baseline-adjusted circulating DPP-4 AUC differed significantly between groups during 0–30 min (F(2,72)=3.91, p=0.024), with 300 mg producing a lower response than placebo (p=0.020); the 150 mg group did not differ significantly from either group. No significant differences were observed during 45 min–2.5 h (F(2,68)=1.53, p=0.225) or 3–4 h (F(2,70)=1.23, p=0.298). During 5–6 h, 300 mg produced a lower response than placebo (p=0.008; F(2,64)=5.20, p=0.008), with the 150 mg group showing a non-significant reduction (p=0.061). During 7–8 h, 300 mg was again lower than placebo (p=0.027; F(2,60)=3.59, p=0.034), with other comparisons non-significant. AUC0–8h showed a trend towards significance between groups (F(2,68)=2.59, p=0.082).

**Figure 4:**
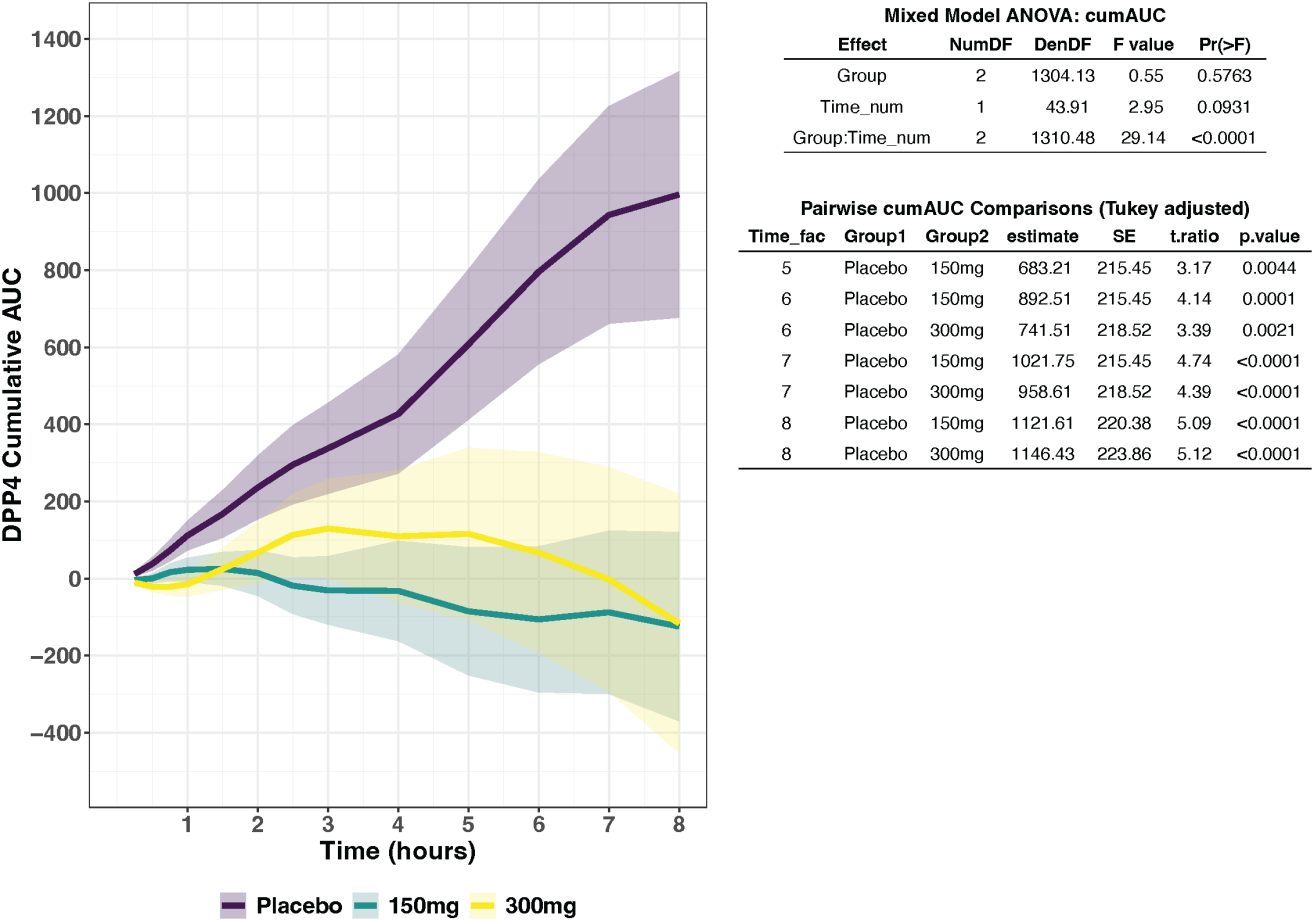
Cumulative AUC of DPP-4 levels over time. Breakfast = time breakfast was consumed, Lunch = time lunch was consumed.

Cumulative AUC analysis of DPP-4 was carried out to assess the differences associated due to TRPTI™ supplementation over time (**Figure 4**). A repeated-measures ANOVA revealed a significant Treatment × Time interaction (p < 0.0001). A significant concentration change of serum DPP-4 was observed with both 150 mg and 300 mg supplementation of TRPTI™ in comparison to placebo, from 5 hours until 8 hours post TRPTI™ supplementation.

**Figure 5:**
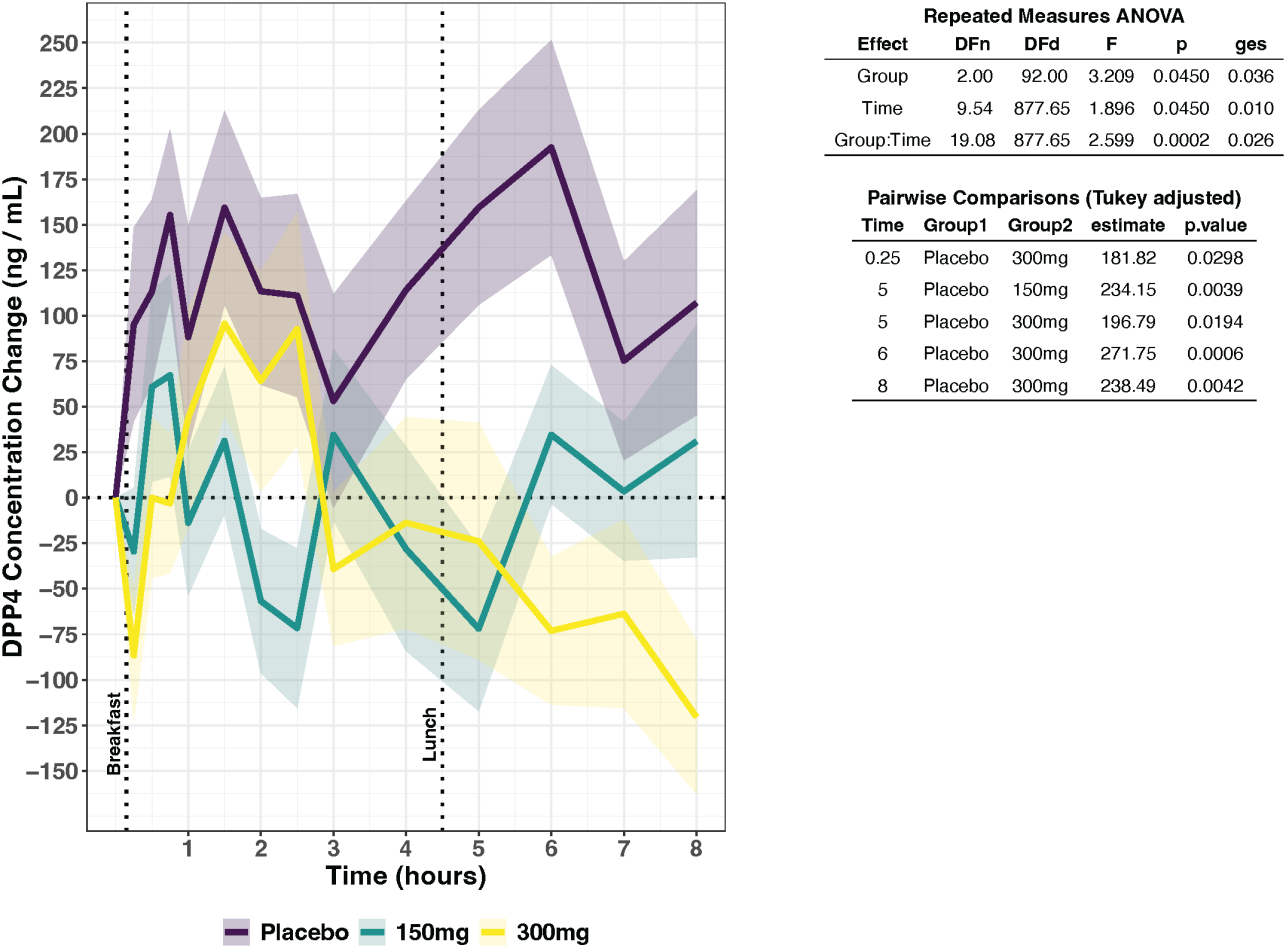
Change in DPP-4 concentration over an 8-hour period following supplementation.

Dose-dependent changes in circulating DPP-4 concentrations were observed across groups (p < 0.05) (**Supplementary Figure 3**). A significant main effect of Treatment was observed for baseline-adjusted DPP-4 concentrations (p < 0.05; **Figure 5**), indicating differences in overall concentrations between treatment conditions. A significant main effect of Time was observed (p < 0.05). A repeated-measures ANOVA also revealed a significant Treatment × Time interaction (p < 0.001). It showed significant reductions at later post-dose timepoints following 300 mg supplementation relative to the placebo. Several timepoints demonstrated statistically significant decreases (p < 0.05). The 150 mg dose produced minimal divergence from placebo.

### 3.5 Glucagon

**Figure 6:**
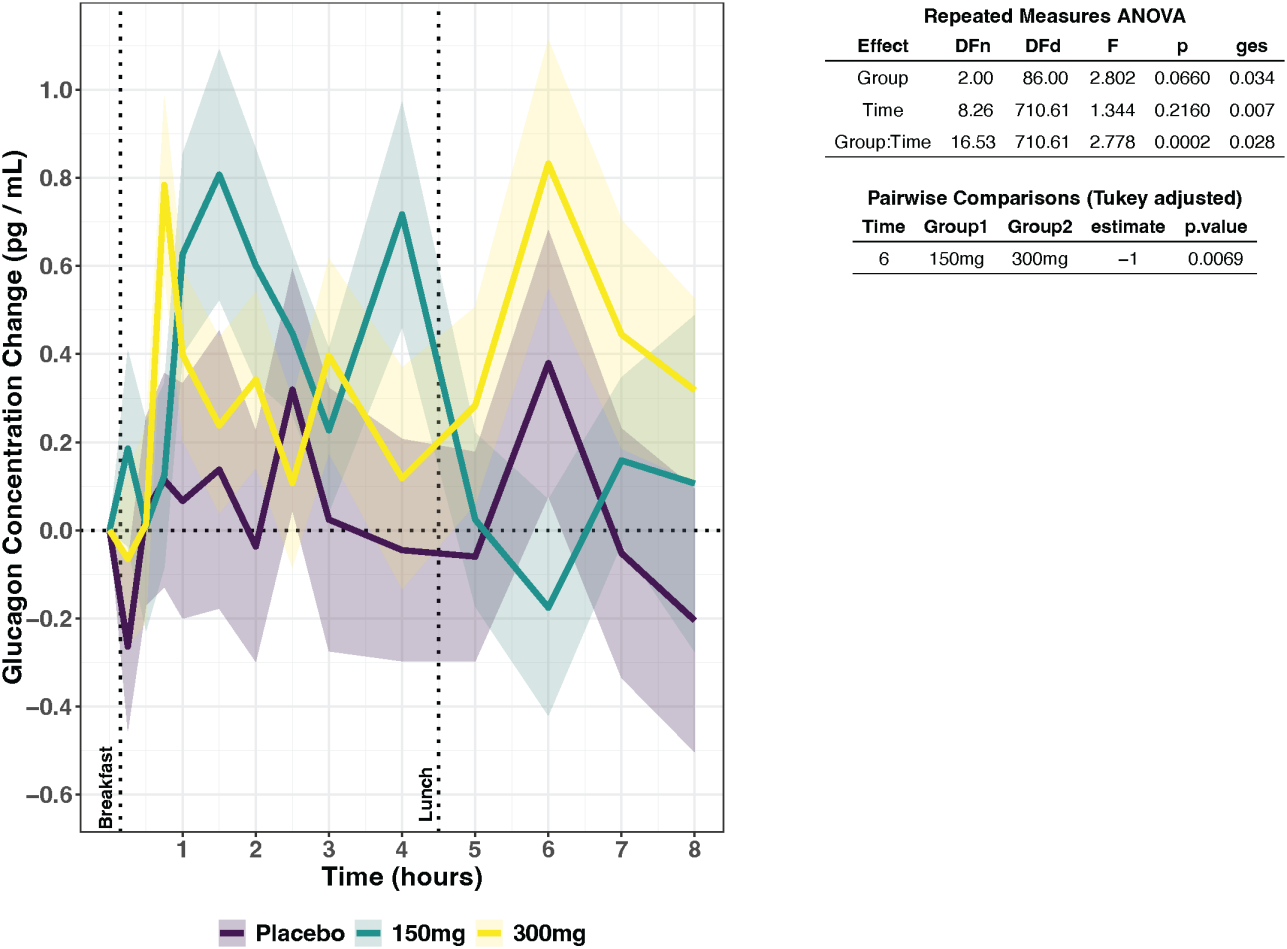
Glucagon concentration over an 8-hour period following supplementation. Change in concentrations over time. Breakfast = time breakfast was consumed, Lunch = time lunch was consumed.

Baseline adjusted glucagon concentrations fluctuated across time in a pattern consistent with physiological post-prandial dynamics (**Figure 6**; **Supplementary Figure 4**). No significant between-group differences were observed for baseline-adjusted glucagon AUC during any prandial period: 0–30 min (F(2,72)=0.57, p=0.569), 45 min–2.5 h (F(2,68)=1.07, p=0.347), 3–4 h (F(2,68)=1.29, p=0.281), 5–6 h (F(2,70)=2.11, p=0.129), or 7–8 h (F(2,48)=1.78, p=0.179). Overall AUC0–8h was similarly non-significant (F(2,68)=0.54, p=0.585), indicating that TRPTI™ supplementation did not alter the integrated glucagon response.

No sustained or dose-dependent differences in glucagon concentration were observed between placebo and either TRPTI™ group. Baseline adjusted glucagon responses were small and inconsistent.

### 3.6 Insulin and glucose

**Figure 7:**
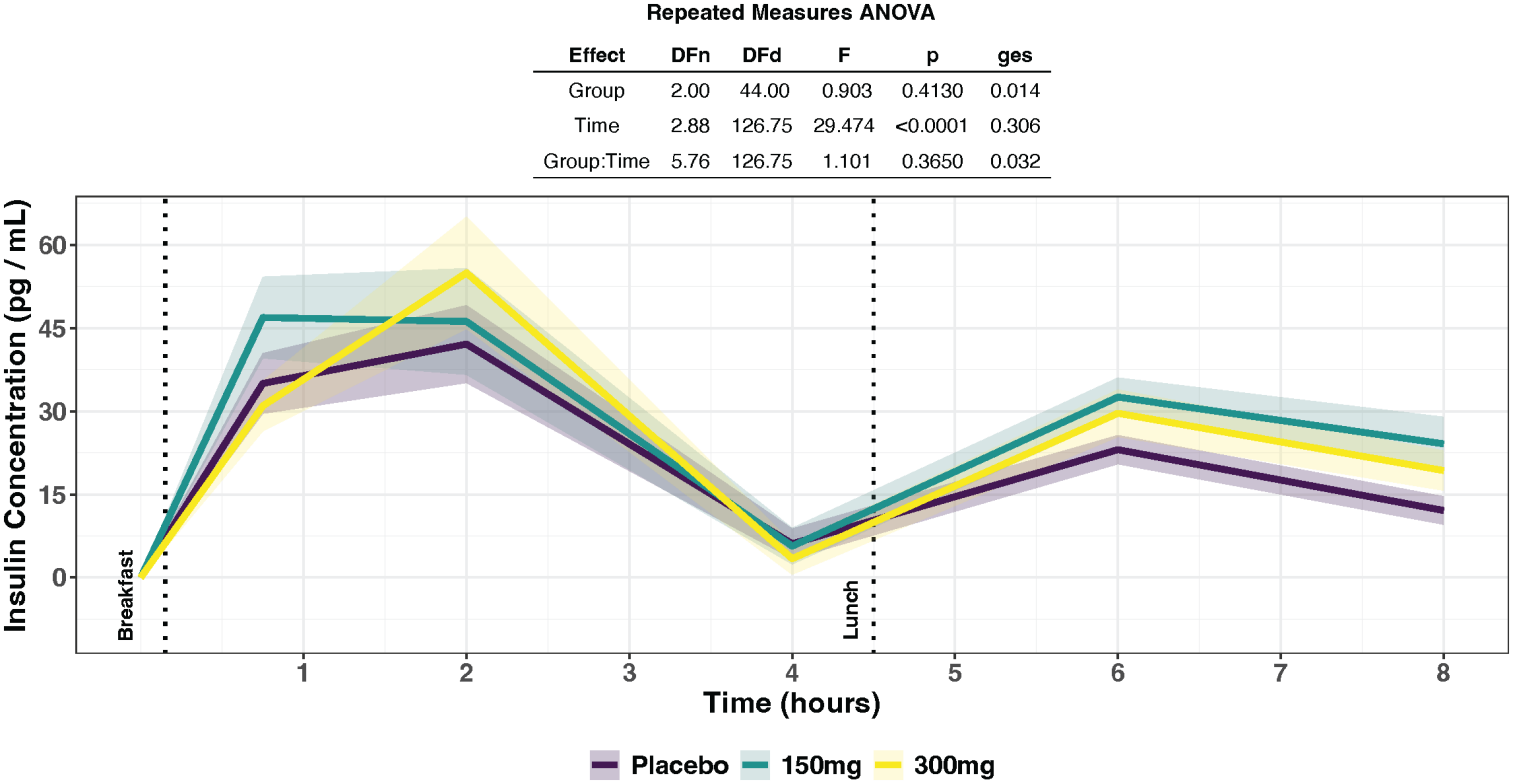
Change in insulin concentration over an 8-hour period following supplementation. Breakfast = time breakfast was consumed, Lunch = time lunch was consumed.

**Figure 8:**
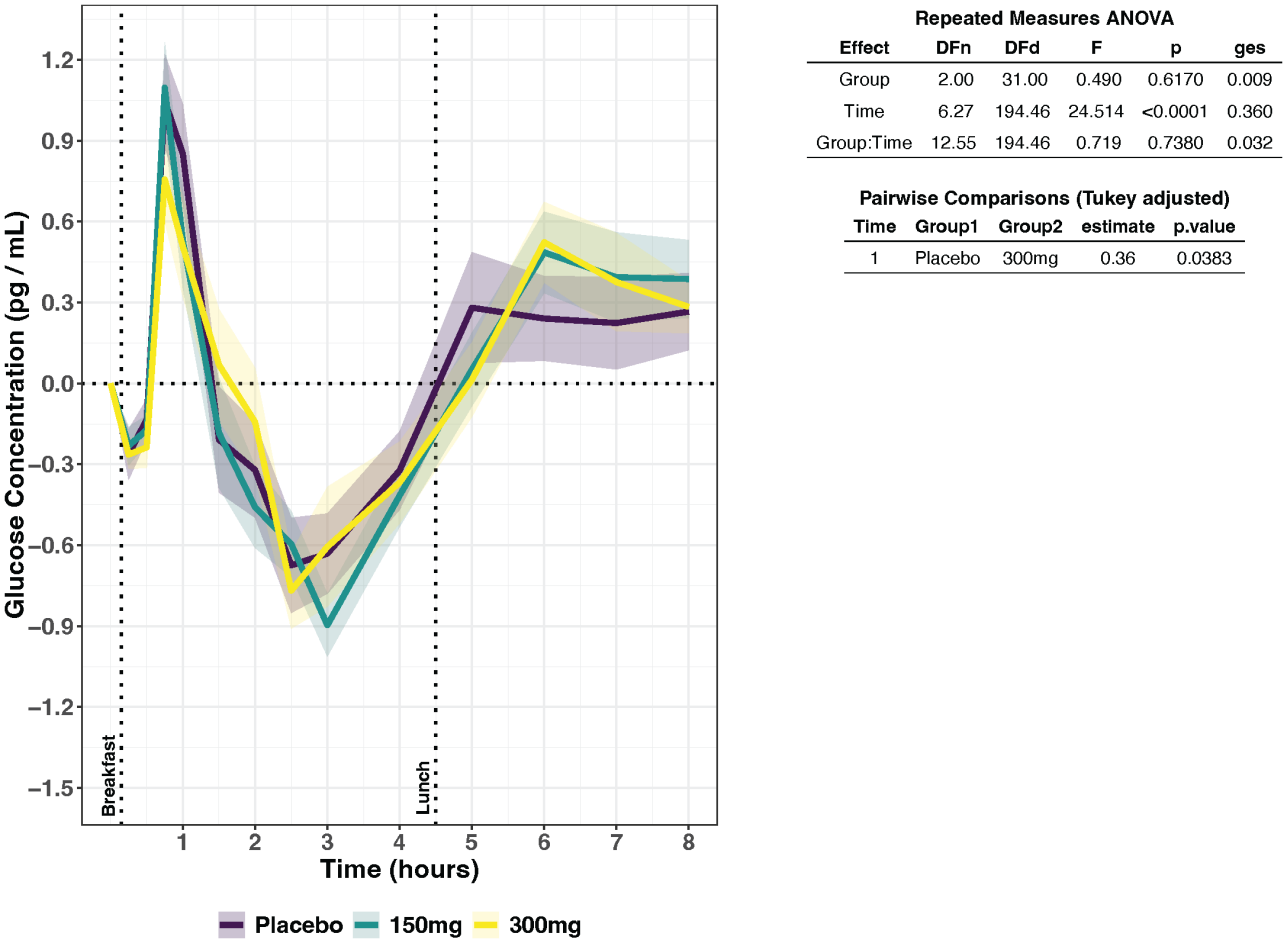
Change in glucose concentrations over an 8-hour period. Breakfast, time breakfast was consumed; Lunch, time lunch was consumed.

Insulin concentrations did not differ between treatment groups at any time point (**Figure 7**; **Supplementary Table 1**). Glucose concentrations followed similar temporal patterns across all groups, with no significant between-group differences at any time point (**Figure 8**; **Supplementary Table 1**).

### 3.7 Meal consumption and appetite/satiety score

Satiety was assessed by means of the VAS before lunch. While the satiety score was consistent between groups, the paired t-test showed that the percentage of the standard lunch meal consumed, was significantly less in the 150 mg TRPTI™ (p = 0.0190) and 300 mg TRPTI™ (p = 0.044) groups compared to placebo (**Table 2**).

**Table 2:** Meal consumption and satiety scores.

Outcome	Placebo n = 37	150 mg TRPTI^TM^ n = 37	300 mg TRPTI^TM^ n = 37
Satiety Score (pre-lunch) mean (SD)	31.8 ± 28.0	31.3 ± 27.3	31.8 ± 23.6
% (SD) of meal remaining – Breakfast	6.1 ± 13.7%	8.8 ± 15.8%	8.8 ± 17.9%
% (SD) of meal remaining – Lunch	6.8 ± 15.2%	14.2 ± 22.5%^a^	11.5 ± 17.3%^a^

SD = standard deviation, a = significant difference (p < 0.05) compared to placebo.

### 3.8 Safety and laboratory parameters

Gastrointestinal tolerability was comparable across treatments, with mild symptoms reported in <5% of participants and no between-group differences observed. Routine clinical chemistry measures performed at baseline and at 8-hours per group, showed only small, timepoint-specific differences, with all values remaining within normal reference ranges. No clinically meaningful treatment-related changes were identified. Detailed laboratory results are provided in **Supplementary Table 1**.

## 4 Discussion

This fixed-sequence, three-period crossover study demonstrates that acute supplementation with TRPTI™ produced a dose-dependent enhancement of incretin responses in healthy adults. Treatment effects were observed under both post-prandial and fasting conditions, suggesting that TRPTI™ potentially augments enteroendocrine signalling as well as altering basal hormone secretion. The 300 mg TRPTI™ dose showed the most consistent biological activity, particularly for serum GLP-1, GIP and DPP-4 concentrations, with no significant treatment-related changes in glucagon.

TRPTI™ exhibited an immediate basal hormone secretion as well as early and sustained post-prandial change between treatments. At 15 minutes post-dose, placebo participants demonstrated a reduction from baseline (−6.4%) and the 150 mg TRPTI™ group displayed a larger reduction (−24.4%), whereas the 300 mg TRPTI™ dose produced a statistically significant increase (+13.6%), corresponding to approximately a 20% elevation relative to placebo. This early difference persisted following the second standardised meal (4 h), with placebo remaining slightly below baseline (−4.6%), the 150 mg TRPTI™ group suppressed (−17.5%), and the 300 mg TRPTI™ group maintaining an elevated GLP-1 response (+20.5%), equating to roughly a 25% greater response than placebo at that timepoint. The consistency of this pattern across repeated meal exposures supports a nutrient-dependent mechanism rather than a transient fluctuation.

The prandial-period AUC analyses provide a framework for interpreting these effects. It should be noted that significant between-group effects were not uniformly driven by differences between 300 mg TRPTI™ and placebo. Rather, several AUC windows reflected lower responses in the 150 mg treatment condition, whereas direct differences between 300 mg TRPTI™ and placebo were evident in only certain periods. Significant between-group differences during the earliest window (0–30 min, pre-prandial) indicate that the 300 mg dose exerted a direct, nutrient-independent stimulatory effect on incretin secretion, consistent with OEA acting via GPR119 on intestinal L- and K-cells prior to meal arrival. The sustained and significant differences during the first post-prandial period (45 min–2.5 h) suggest that TRPTI™ potentiates the meal-stimulated incretin response, amplifying the normal GLP-1 and GIP secretory burst triggered by breakfast. Significant differences during the 3–4 h pre-prandial window and the 5–6 h post-lunch period further indicate that the effect is not confined to a single meal exposure but recurs across repeated nutrient stimuli, consistent with ongoing GPR119 engagement throughout the gastrointestinal transit of the dose. The persistence of between-group differences into the 7–8 h window for GIP, but not GLP-1, may reflect differential rates of clearance or L- versus K-cell sensitivity across the distal gut. Collectively, the prandial-period AUC pattern supports a model in which TRPTI™ primes enteroendocrine cells for enhanced secretion both independently of and in concert with nutrient ingestion.

GLP-1 is secreted from intestinal L-cells in response to macronutrient exposure and enhances glucose-dependent insulin secretion whilst modulating gastric emptying and satiety (13–15). Preclinical evidence identifies OEA as an activator of both PPAR-α and GPR119, mechanisms linked to enteroendocrine signalling and incretin secretion (16–18). The present study extends this mechanistic framework by demonstrating that acute oral TRPTI™ augments meal-stimulated GLP-1 responses under controlled feeding conditions.

Clinical trials of other natural supplements have often shown inconsistent effects on GLP-1. Obese individuals supplemented with resveratrol did not significantly alter fasting or post-prandial GLP-1 despite mechanistic rationale (18), and systematic evaluations of dietary supplements report limited evidence for robust GLP-1 enhancement (19). In this context, the timepoint-specific GLP-1 augmentation observed with TRPTI™, particularly during post-prandial periods, represents a meaningful biological signal.

GIP responses paralleled GLP-1 dynamics. The 300 mg dose produced greater positive deviations from placebo during early and later post-prandial periods, with percentage differences generally ranging from ~15–25% at peak timepoints. GIP, co-secreted with GLP-1 from enteroendocrine K- and L-cells, contributes to post-prandial insulin secretion and metabolic homeostasis (10, 20). The coordinated enhancement of both incretins suggests a generalised potentiation of nutrient-responsive enteroendocrine activity rather than an isolated hormonal fluctuation.

DPP-4 concentrations were relatively stable over time but demonstrated late-phase concentration changes in the 300 mg TRPTI™ group (approximately 10–20% relative to placebo at key timepoints). Studies have shown that pharmacological DPP-4 inhibition enhances incretin bioavailability and improves glycaemic control in type 2 diabetes (13, 21). Although robust human evidence for meaningful modulation of DPP-4 through nutritional intervention remains limited and is largely derived from preclinical or *in vitro* work (19), the directionally consistent reductions in circulating DPP-4 concentrations observed here suggests that TRPTI™ may influence DPP-4 regulation. However, the functional significance of these changes remains to be established. Accordingly, the present findings should be interpreted as evidence of altered circulating DPP-4 concentrations rather than direct evidence of altered DPP-4 activity or incretin hormone degradation. Future studies incorporating direct DPP-4 activity measures or active hormone kinetics will be required to confirm functional relevance.

Glucagon followed expected post-prandial dynamics and did not differ consistently between treatments. The absence of over-suppression or dysregulation of glucagon indicates that TRPTI’s™ incretin modulation does not perturb counter-regulatory hormonal balance in healthy adults. Prior supplementation trials (e.g., resveratrol) have also demonstrated negligible effects on glucagon despite broad metabolic interest (18).

Both insulin and glucose concentrations exhibited similar temporal patterns across all groups, with no significant between-group differences at any timepoint, indicating no change despite modulation of upstream incretin signalling. This observation suggests that although TRPTI™ influenced GLP-1, GIP, and DPP-4 responses, the magnitude and duration of incretin stimulation achieved following a single dose was insufficient to produce measurable changes in downstream glycaemic regulation under the conditions of the present study. Importantly, this study was designed as an acute pharmacodynamic investigation to determine whether TRPTI™ could engage incretin-related pathways, rather than to evaluate clinical efficacy or glycaemic outcomes. Given the transient nature of the observed hormonal responses and the healthy metabolic status of the study population, the absence of changes in insulin or glucose is not unexpected. Whether repeated administration can amplify or sustain these endocrine effects sufficiently to influence metabolic outcomes remains to be determined in longer-duration studies.

Pre-lunch satiety scores assessed by visual analogue scale were comparable across groups, but both TRPTI™ groups consumed a lower percentage of the standardised meal compared with placebo, particularly at lunch. Although differences were modest and subjective ratings were similar, the directional reduction in energy intake aligns with rodent and limited human evidence implicating OEA pathways in satiety and feeding regulation (16, 22). In animal models, OEA administration reduces meal size and prolongs inter-meal intervals via PPAR-α–mediated pathways and vagal sensory signalling (22, 23), although translation to human appetite regulation is less consistently reported and a direction for future studies.

TRPTI™ was well tolerated, with gastrointestinal symptoms reported in fewer than 5% of participants and no meaningful between-group differences. Routine clinical chemistry parameters, including liver function tests (LFTs), remained within normal reference ranges throughout the study, with only minor timepoint-specific fluctuations and no evidence of clinically significant treatment-related changes. These findings are consistent with the safety profile observed in prior human incretin and lipid signalling studies, where short-term nutritional modulation of enteroendocrine pathways did not produce adverse hepatic or systemic laboratory changes (18, 20, 24). The absence of acute adverse signals supports tolerability of single-dose TRPTI™ administration at both tested doses.

Although the present study provides mechanistic insight into acute incretin modulation, several limitations warrant consideration. Significantly elevated pre-dose GLP-1 and GIP concentrations were observed at the 150 mg visit relative to both the placebo and 300 mg visits. Because all analyses were baseline-adjusted by subtracting the 0 h value, a higher pre-dose starting point mathematically compresses the post-dose trajectory relative to placebo, and in several prandial windows produced an artefactual suppression pattern in which the 150 mg group appeared to perform below placebo. This is most likely attributable to period or order effects inherent to the fixed-sequence design, in which all participants received treatments in the same predefined order, rather than a genuine pharmacological suppression of incretin secretion by the 150 mg dose. The study adopted this design because the absorption profile and duration of action of TRPTI™ had not previously been characterised in humans, and therefore the potential for carryover effects could not be confidently excluded during study planning. A minimum 6-day washout was incorporated between treatment periods to minimise carryover, but because the absorption and elimination profile of TRPTI™ had not been previously characterised in humans, residual biological or physiological carry-in effects between the placebo and 150 mg visits cannot be entirely excluded. Consequently, the apparent underperformance of the 150 mg dose relative to placebo should be interpreted cautiously and does not necessarily reflect the true pharmacological potential of this dose. Circulating DPP-4 concentrations were measured rather than enzymatic activity, and total rather than active incretin fractions were assessed, limiting mechanistic specificity. Consequently, the functional significance of the observed reductions in DPP-4 concentration on incretin preservation cannot be determined from the present study and warrants further investigation. Therefore, longer-duration trials incorporating enzymatic activity measures and validated appetite assessments are warranted.

Standardised meals were provided at each visit, and participants consumed this food ad libitum to avoid introducing bias associated with compulsory overconsumption. Consequently, the absolute quantity of food consumed may have varied both between study visits within the same participant and/or between participants for the same visit. However, meal composition and timing were identical across treatment visits, and the change in consumption volumes is unlikely to alter incretin hormones significantly. Additionally, the observed reductions in food intake following TRPTI™ administration are themselves consistent with the proposed satiety-related mechanisms of OEA. Therefore, whilst variation in meal quantity cannot be completely excluded as a contributing factor, it is unlikely to fully account for the consistent treatment-related differences observed in the incretin hormone responses.

The primary outcome of GLP-1 AUC analyses demonstrated significant between-group differences across all five meal-related windows, with time-course analyses providing further characterisation of the temporal pattern of these effects. Together, these findings demonstrate rapid and measurable incretin modulation following a single dose of TRPTI™, providing preliminary proof-of-mechanism. Although the magnitude of the acute hormonal response was insufficient to influence insulin or glucose concentration in the current study. Chronic incretin modulation strategies such as GLP-1 receptor agonists and DPP-4 inhibitors have produced substantial metabolic benefits in obesity and type 2 diabetes (13, 25). The observable timepoint-specific endocrine effects following a single 300 mg TRPTI™ dose provides rationale for longer-duration supplementation studies to determine whether repeated administration amplifies effect magnitude, prolongs duration, or translates into improvements in glycaemic regulation, appetite control, or metabolic health.

Future investigations should determine whether repeated administration of TRPTI™ amplifies effect magnitude, prolongs post-prandial GLP-1 and GIP elevation, modulates DPP-4 levels, or translates into clinically meaningful improvements in glycaemic regulation, appetite control, body weight, or metabolic health. Given that incretin responsiveness and DPP-4 dynamics differ in individuals with insulin resistance or impaired glucose tolerance, extending this work to metabolically at-risk populations may be of particularly interest.

## 5 Conclusions

In summary, acute supplementation with TRPTI™ enhances meal-stimulated total GLP-1 and GIP responses in a dose-dependent manner, with late-phase changes in circulating DPP-4 concentrations. Insulin and glucose remained equivalent between groups, and treatment was well tolerated. Effects were prominent at post-meal periods, consistent with a nutrient-dependent mechanism of action. Compared with many natural bioactive compounds that have produced inconsistent incretin effects in humans, TRPTI™ demonstrated reproducible time-specific GLP-1 and GIP increases following a single dose, providing a foundation for future longer-term investigations.

## Data Availability

The raw data supporting the conclusions of this article will be made available by the authors, without undue reservation.
