# Supplementary material for: Acute TRPTI™ (oleoylethanolamide) supplementation enhances incretin hormone responses: a fixed-sequence crossover study
Source: Front Endocrinol (Lausanne). 2026 Jul 15;17:1879799. doi: 10.3389/fendo.2026.1879799 (PMC13414813; doi:10.3389/fendo.2026.1879799)
Supplement: Supplementary file 1 [file DataSheet1.docx]

**Supplementary Data**

**Table S1.** Safety, Metabolic and Biochemical Parameters Following Placebo, 150 mg and 300 mg TRPTI™. Values are presented as mean ± SD. p-values represent pairwise comparisons (1 vs 2, 1 vs 3, 2 vs 3).

**Glucose (mmol/L)**

| Time (h) | Placebo | 150 mg TRPTI^TM^ | 300 mg TRPTI^TM^ | 1 vs 2 | 1 vs 3 | 2 vs 3 |
| --- | --- | --- | --- | --- | --- | --- |
| 0.0 | 5.22 ± 0.79 | 5.28 ± 0.86 | 5.30 ± 1.04 | 0.748 | 0.688 | 0.911 |
| 0.25 | 4.84 ± 0.53 | 4.92 ± 0.34 | 4.81 ± 0.32 | 0.558 | 0.848 | 0.298 |
| 0.5 | 5.13 ± 0.96 | 5.18 ± 0.98 | 5.19 ± 1.24 | 0.845 | 0.832 | 0.968 |
| 0.75 | 6.27 ± 1.24 | 6.38 ± 1.18 | 6.06 ± 1.38 | 0.714 | 0.504 | 0.302 |
| 1.0 | 6.06 ± 1.46 | 5.84 ± 1.45 | 5.81 ± 1.66 | 0.529 | 0.511 | 0.950 |
| 1.5 | 5.00 ± 1.64 | 5.00 ± 1.24 | 5.37 ± 1.84 | 0.992 | 0.382 | 0.330 |
| 2.0 | 4.90 ± 1.57 | 4.72 ± 0.96 | 5.17 ± 1.99 | 0.568 | 0.538 | 0.250 |
| 2.5 | 4.41 ± 1.03 | 4.53 ± 0.60 | 4.35 ± 0.79 | 0.543 | 0.827 | 0.309 |
| 3.0 | 4.59 ± 1.32 | 4.38 ± 1.19 | 4.70 ± 2.12 | 0.495 | 0.786 | 0.437 |
| 4.0 | 4.97 ± 0.79 | 4.91 ± 0.93 | 4.99 ± 1.74 | 0.784 | 0.938 | 0.803 |
| 5.0 | 5.57 ± 0.80 | 5.41 ± 1.02 | 5.36 ± 1.46 | 0.539 | 0.533 | 0.891 |
| 6.0 | 5.49 ± 0.73 | 5.80 ± 1.20 | 5.89 ± 1.38 | 0.234 | 0.160 | 0.783 |
| 7.0 | 5.33 ± 0.51 | 5.54 ± 0.65 | 5.61 ± 0.81 | 0.244 | 0.174 | 0.740 |
| 8.0 | 5.30 ± 0.52 | 5.53 ± 0.67 | 5.41 ± 0.42 | 0.182 | 0.444 | 0.473 |

**Total Protein (g/L)**

| Time (h) | Placebo | 150 mg TRPTI^TM^ | 300 mg TRPTI^TM^ | 1 vs 2 | 1 vs 3 | 2 vs 3 |
| --- | --- | --- | --- | --- | --- | --- |
| 0.0 | 64.07 ± 5.25 | 63.30 ± 4.96 | 62.43 ± 5.19 | 0.522 | 0.187 | 0.473 |
| 0.75 | 61.75 ± 5.01 | 61.87 ± 4.62 | 62.17 ± 5.23 | 0.918 | 0.732 | 0.797 |
| 2.0 | 62.31 ± 5.62 | 62.04 ± 4.99 | 61.25 ± 4.78 | 0.834 | 0.400 | 0.513 |
| 4.0 | 62.39 ± 5.87 | 62.75 ± 5.91 | 61.94 ± 5.56 | 0.809 | 0.758 | 0.574 |
| 6.0 | 64.34 ± 6.17 | 65.45 ± 7.73 | 65.21 ± 6.81 | 0.543 | 0.602 | 0.902 |
| 8.0 | 64.02 ± 7.21 | 66.59 ± 8.28 | 67.13 ± 8.28 | 0.248 | 0.167 | 0.828 |

**Albumin (g/L)**

| Time (h) | Placebo | 150 mg TRPTI^TM^ | 300 mg TRPTI^TM^ | 1 vs 2 | 1 vs 3 | 2 vs 3 |
| --- | --- | --- | --- | --- | --- | --- |
| 0.0 | 43.42 ± 2.00 | 42.90 ± 1.99 | 42.43 ± 2.17 | 0.273 | 0.048 | 0.342 |
| 0.75 | 42.11 ± 2.23 | 42.23 ± 1.72 | 42.38 ± 1.71 | 0.806 | 0.573 | 0.712 |
| 2.0 | 42.29 ± 2.06 | 41.99 ± 1.82 | 42.08 ± 1.53 | 0.530 | 0.634 | 0.824 |
| 4.0 | 42.08 ± 2.98 | 42.15 ± 2.02 | 42.05 ± 1.77 | 0.915 | 0.952 | 0.825 |
| 6.0 | 42.63 ± 1.82 | 42.63 ± 2.00 | 42.53 ± 1.86 | 1.000 | 0.829 | 0.841 |
| 8.0 | 42.98 ± 2.61 | 43.57 ± 2.04 | 43.23 ± 2.48 | 0.379 | 0.735 | 0.611 |

**Alanine Aminotransferase (ALT; U/L)**

| Time (h) | Placebo | 150 mg TRPTI^TM^ | 300 mg TRPTI^TM^ | 1 vs 2 | 1 vs 3 | 2 vs 3 |
| --- | --- | --- | --- | --- | --- | --- |
| 0.0 | 15.91 ± 7.09 | 16.23 ± 7.72 | 15.17 ± 6.53 | 0.857 | 0.648 | 0.534 |
| 0.75 | 15.30 ± 6.49 | 15.77 ± 7.71 | 16.02 ± 7.37 | 0.783 | 0.664 | 0.892 |
| 2.0 | 16.98 ± 7.46 | 17.94 ± 8.43 | 16.89 ± 7.28 | 0.624 | 0.958 | 0.589 |
| 4.0 | 18.62 ± 7.81 | 18.76 ± 10.05 | 17.59 ± 7.80 | 0.950 | 0.606 | 0.602 |
| 6.0 | 18.58 ± 6.15 | 21.18 ± 8.25 | 20.81 ± 7.18 | 0.178 | 0.209 | 0.859 |
| 8.0 | 18.23 ± 7.38 | 20.71 ± 11.13 | 19.74 ± 7.46 | 0.357 | 0.492 | 0.737 |

**Aspartate Aminotransferase (AST; U/L)**

| Time (h) | Placebo | 150 mg TRPTI^TM^ | 300 mg TRPTI^TM^ | 1 vs 2 | 1 vs 3 | 2 vs 3 |
| --- | --- | --- | --- | --- | --- | --- |
| 0.0 | 21.92 ± 7.31 | 22.44 ± 7.71 | 21.95 ± 8.42 | 0.770 | 0.989 | 0.796 |
| 0.75 | 21.31 ± 7.03 | 22.97 ± 7.84 | 23.03 ± 8.37 | 0.357 | 0.352 | 0.977 |
| 2.0 | 24.26 ± 7.94 | 24.14 ± 7.07 | 24.37 ± 8.54 | 0.951 | 0.956 | 0.908 |
| 4.0 | 25.85 ± 7.65 | 25.95 ± 7.96 | 25.33 ± 8.79 | 0.958 | 0.808 | 0.768 |
| 6.0 | 27.35 ± 8.08 | 29.06 ± 7.08 | 30.85 ± 8.80 | 0.395 | 0.120 | 0.406 |
| 8.0 | 24.72 ± 7.14 | 27.90 ± 9.18 | 27.03 ± 7.81 | 0.179 | 0.295 | 0.738 |

**Cholesterol (CHO; mmol/L)**

| Time (h) | Placebo | 150 mg TRPTI^TM^ | 300 mg TRPTI^TM^ | 1 vs 2 | 1 vs 3 | 2 vs 3 |
| --- | --- | --- | --- | --- | --- | --- |
| 0.0 | 5.39 ± 1.07 | 5.43 ± 1.04 | 5.33 ± 0.98 | 0.870 | 0.814 | 0.682 |
| 0.75 | 5.17 ± 1.02 | 5.25 ± 0.98 | 5.30 ± 0.94 | 0.761 | 0.597 | 0.822 |
| 2.0 | 5.20 ± 1.06 | 5.17 ± 0.99 | 5.25 ± 0.87 | 0.908 | 0.828 | 0.729 |
| 4.0 | 5.12 ± 1.12 | 5.13 ± 0.99 | 5.15 ± 0.90 | 0.964 | 0.914 | 0.947 |
| 6.0 | 5.30 ± 0.99 | 5.27 ± 1.03 | 5.46 ± 1.02 | 0.931 | 0.538 | 0.502 |
| 8.0 | 5.54 ± 0.92 | 5.63 ± 0.97 | 5.55 ± 0.96 | 0.744 | 0.965 | 0.793 |

**Creatinine (umol/L)**

| Time (h) | Placebo | 150 mg TRPTI^TM^ | 300 mg TRPTI^TM^ | 1 vs 2 | 1 vs 3 | 2 vs 3 |
| --- | --- | --- | --- | --- | --- | --- |
| 0.0 | 81.43 ± 17.98 | 81.30 ± 15.23 | 82.21 ± 15.42 | 0.973 | 0.845 | 0.802 |
| 0.75 | 79.25 ± 17.37 | 78.55 ± 16.23 | 79.90 ± 14.83 | 0.862 | 0.865 | 0.714 |
| 2.0 | 76.66 ± 15.65 | 78.30 ± 14.84 | 77.61 ± 14.27 | 0.661 | 0.792 | 0.848 |
| 4.0 | 72.85 ± 17.44 | 74.37 ± 15.00 | 76.22 ± 16.00 | 0.708 | 0.427 | 0.633 |
| 6.0 | 77.06 ± 18.01 | 78.04 ± 20.34 | 79.06 ± 20.25 | 0.845 | 0.685 | 0.849 |
| 8.0 | 75.37 ± 15.90 | 83.78 ± 20.47 | 79.40 ± 20.33 | 0.109 | 0.440 | 0.476 |

**Gamma-Glutamyl Transferase (GGT; U/L)**

| Time (h) | Placebo | 150 mg TRPTI^TM^ | 300 mg TRPTI^TM^ | 1 vs 2 | 1 vs 3 | 2 vs 3 |
| --- | --- | --- | --- | --- | --- | --- |
| 0.0 | 19.36 ± 12.08 | 18.94 ± 10.71 | 18.89 ± 10.58 | 0.877 | 0.860 | 0.982 |
| 0.75 | 19.20 ± 11.46 | 18.70 ± 10.17 | 18.36 ± 9.54 | 0.849 | 0.738 | 0.888 |
| 2.0 | 17.86 ± 11.21 | 17.47 ± 9.81 | 18.82 ± 11.71 | 0.881 | 0.727 | 0.613 |
| 4.0 | 18.20 ± 12.01 | 17.42 ± 10.44 | 18.97 ± 11.96 | 0.785 | 0.802 | 0.581 |
| 6.0 | 15.69 ± 8.91 | 16.14 ± 9.16 | 15.50 ± 10.34 | 0.851 | 0.940 | 0.804 |
| 8.0 | 16.68 ± 12.07 | 16.70 ± 11.16 | 17.59 ± 11.28 | 0.996 | 0.791 | 0.790 |

**Total Bilirubin (µmol/L)**

| Time (h) | Placebo | 150 mg TRPTI^TM^ | 300 mg TRPTI^TM^ | 1 vs 2 | 1 vs 3 | 2 vs 3 |
| --- | --- | --- | --- | --- | --- | --- |
| 0.0 | 15.29 ± 8.32 | 14.56 ± 7.75 | 14.84 ± 8.33 | 0.701 | 0.818 | 0.884 |
| 0.75 | 15.38 ± 8.15 | 15.38 ± 7.24 | 15.77 ± 8.18 | 0.999 | 0.840 | 0.831 |
| 2.0 | 14.95 ± 7.75 | 15.03 ± 7.41 | 15.49 ± 7.97 | 0.968 | 0.780 | 0.810 |
| 4.0 | 13.64 ± 7.17 | 13.83 ± 6.62 | 13.83 ± 7.87 | 0.909 | 0.921 | 0.996 |
| 6.0 | 13.13 ± 7.28 | 13.67 ± 7.11 | 13.61 ± 8.09 | 0.772 | 0.808 | 0.974 |
| 8.0 | 12.55 ± 6.96 | 13.99 ± 6.05 | 12.03 ± 7.00 | 0.443 | 0.799 | 0.325 |

**Triglycerides (mmol/L)**

| Time (h) | Placebo | 150 mg TRPTI^TM^ | 300 mg TRPTI^TM^ | 1 vs 2 | 1 vs 3 | 2 vs 3 |
| --- | --- | --- | --- | --- | --- | --- |
| 0.0 | 1.16 ± 0.75 | 1.19 ± 0.82 | 1.08 ± 0.67 | 0.892 | 0.638 | 0.554 |
| 0.75 | 1.13 ± 0.74 | 1.13 ± 0.73 | 1.11 ± 0.70 | 0.987 | 0.887 | 0.899 |
| 2.0 | 1.42 ± 0.93 | 1.40 ± 0.77 | 1.40 ± 0.80 | 0.949 | 0.929 | 0.977 |
| 4.0 | 1.70 ± 1.12 | 1.90 ± 1.19 | 1.75 ± 1.14 | 0.487 | 0.862 | 0.600 |
| 6.0 | 2.26 ± 1.40 | 2.59 ± 1.58 | 2.42 ± 1.37 | 0.392 | 0.652 | 0.657 |
| 8.0 | 1.96 ± 1.18 | 2.35 ± 1.49 | 2.51 ± 1.62 | 0.314 | 0.181 | 0.730 |

**Insulin (µIU/mL)**

| Time  (h) | Placebo | 150 mg TRPTI^TM^ | 300 mg TRPTI^TM^ | 1 vs 2 | 1 vs 3 | 2 vs 3 |
| --- | --- | --- | --- | --- | --- | --- |
| 0.0 | 11.29 ± 14.02 | 8.52 ± 6.30 | 9.39 ± 9.83 | 0.282 | 0.506 | 0.657 |
| 0.75 | 46.56 ± 35.14 | 55.42 ± 48.48 | 40.34 ± 27.06 | 0.382 | 0.406 | 0.108 |
| 2.0 | 53.64 ± 43.73 | 54.48 ± 56.85 | 63.59 ± 63.02 | 0.946 | 0.446 | 0.538 |
| 4.0 | 18.46 ± 19.29 | 16.62 ± 16.76 | 15.63 ± 16.56 | 0.685 | 0.534 | 0.811 |
| 6.0 | 32.83 ± 15.75 | 41.23 ± 21.31 | 39.48 ± 26.63 | 0.088 | 0.238 | 0.784 |
| 8.0 | 21.80 ± 13.63 | 31.18 ± 23.92 | 27.03 ± 19.22 | 0.089 | 0.271 | 0.526 |


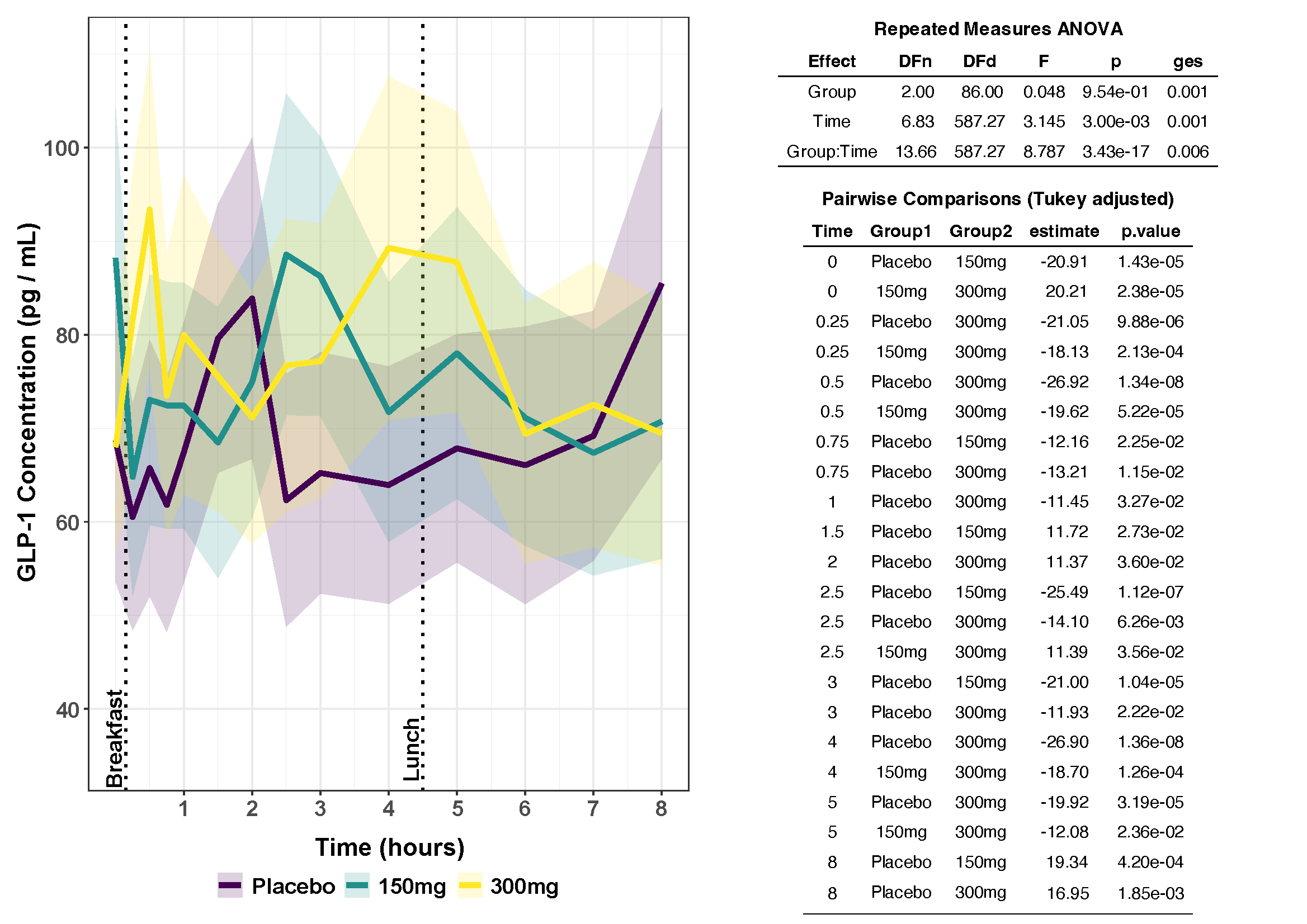


**Figure S1.** Absolute GLP-1 Concentration over Time

**
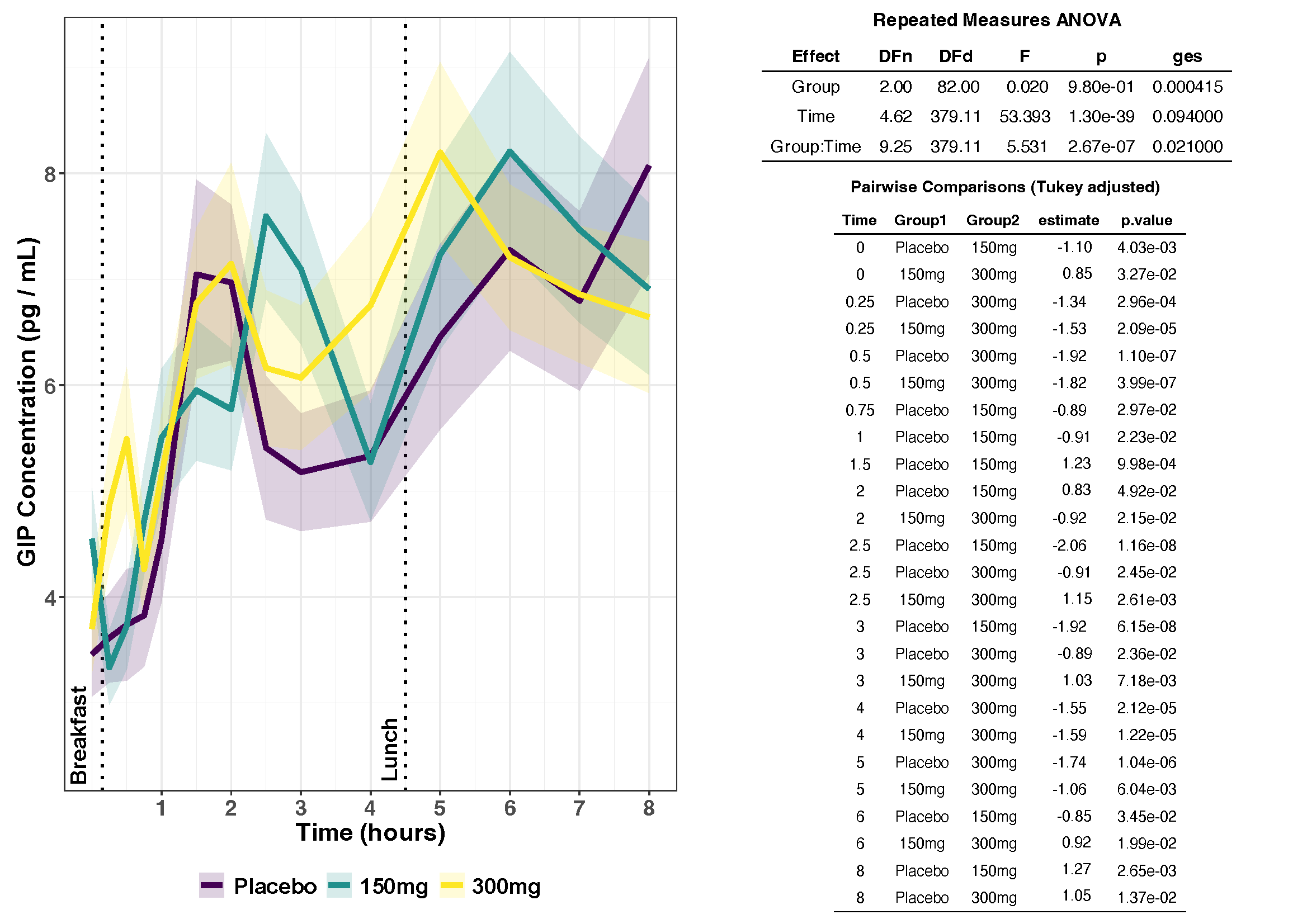
**

**Figure S2.** Absolute GIP Concentration over Time

**
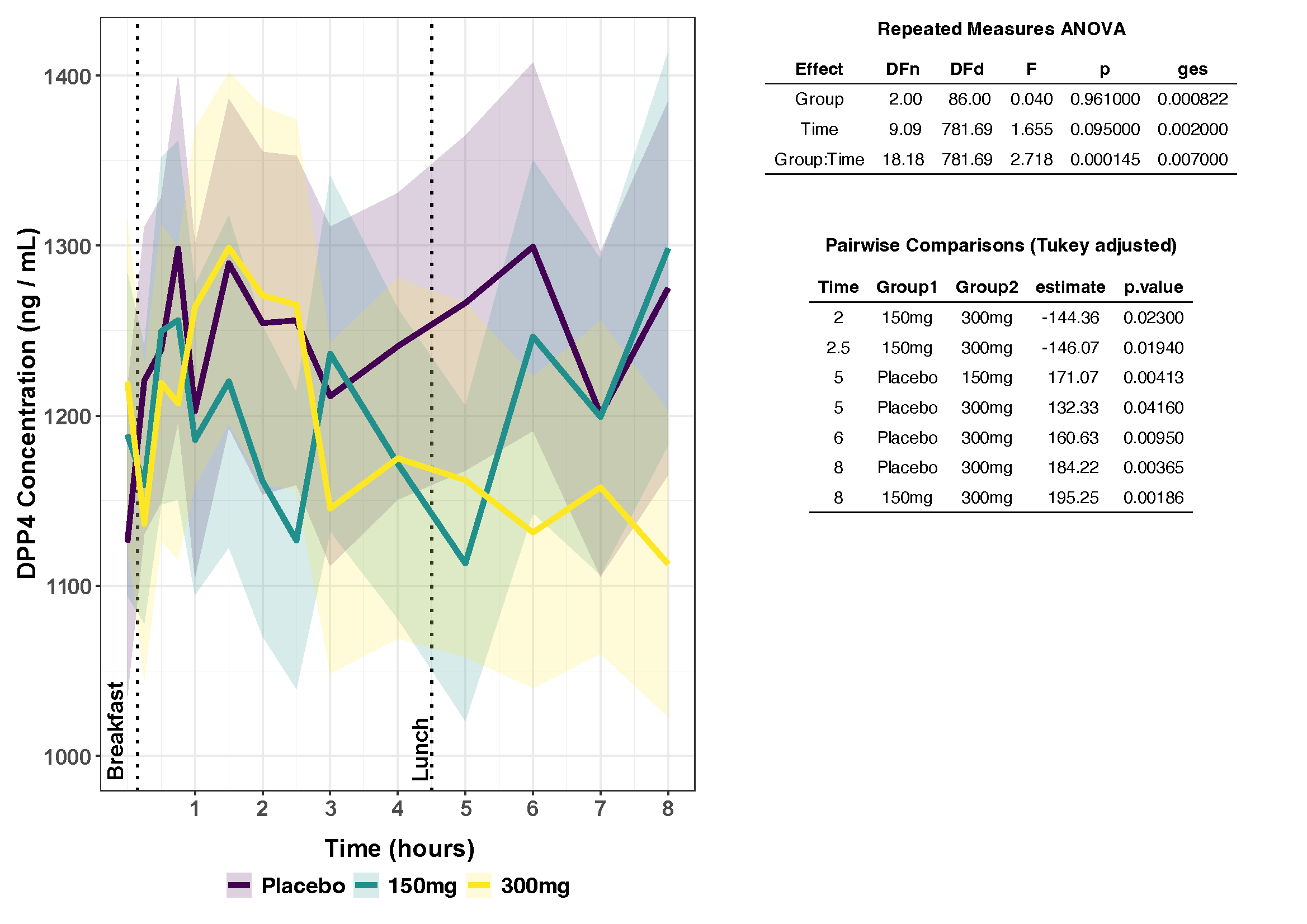
**

**Figure S3.** Absolute DPP-4 Concentration over Time

**
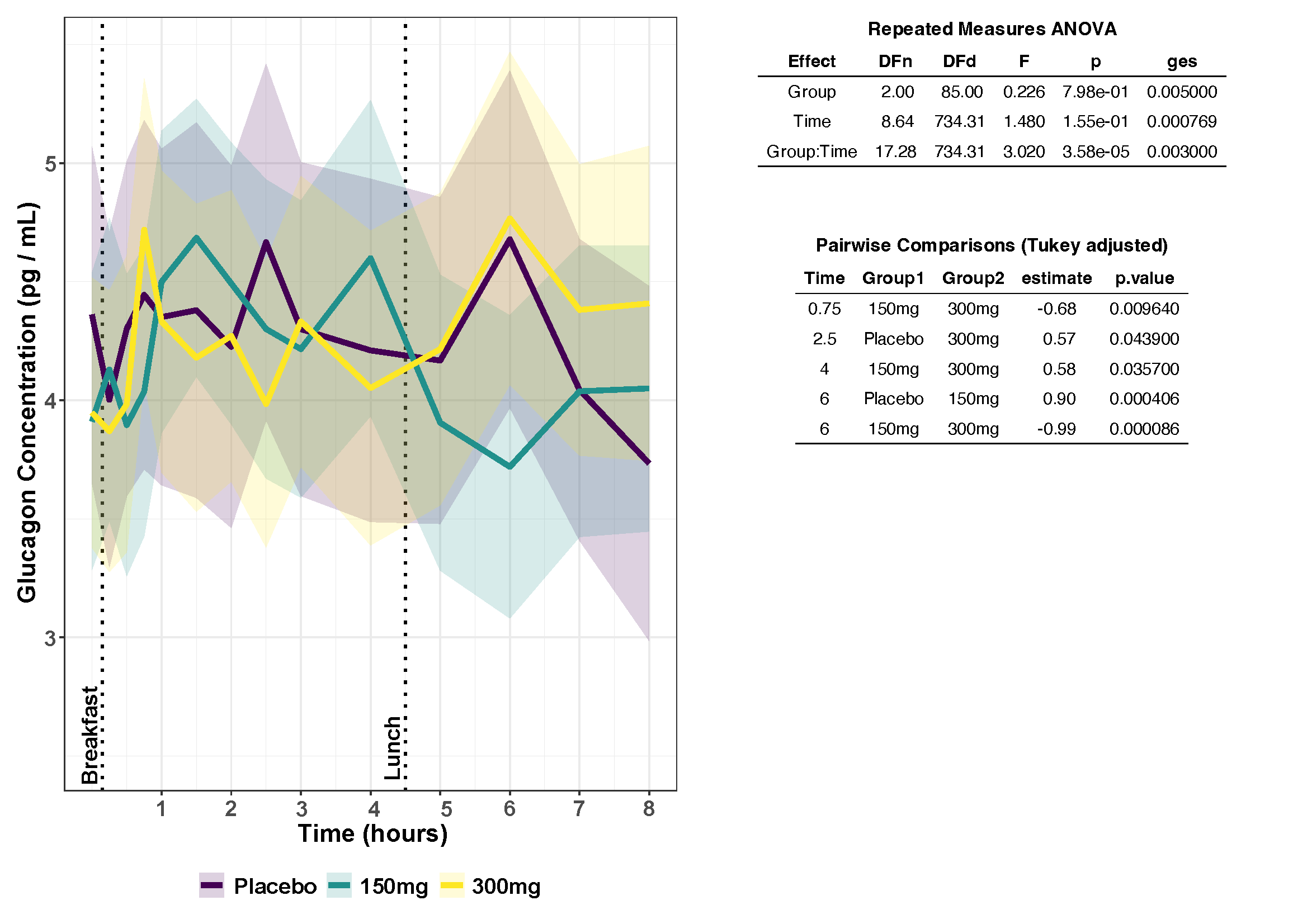
**

**Figure S4.** Absolute Glucagon Concentration over Time
